# The new abnormal: Identifying and ranking anomalies in the land trade market

**DOI:** 10.1371/journal.pone.0277608

**Published:** 2022-12-01

**Authors:** Roberto Interdonato, Jeremy Bourgoin, Quentin Grislain, Andrea Tagarelli

**Affiliations:** 1 CIRAD, UMR TETIS, Montpellier, France; 2 TETIS, Univ. Montpellier, AgroParisTech, CIRAD, CNRS, INRAE, Montpellier, France; 3 DIMES, University of Calabria, Arcavacata, Italy; Unviersity of Burgundy, FRANCE

## Abstract

Large-scale national and transnational commercial land transactions, or Large-Scale Land Acquisitions (LSLAs), have been gaining a lot of academic attention since the late 2000s and since the reported rush for land, resulting in turn from an increase in demand for arable land. If many data exist to characterize land deals, the analysis of investment networks remain limited and predominantly portrays power asymmetries between countries from the Global North investing in the Global South. The aim of this work is to perform a deeper investigation on the land trade market, specifically focusing on cases that do not follow such narratives. For instance, almost 25% of the countries included in the transnational land trade network do not follow a strict investor/target dichotomy, thus being characterized by a double role, i.e., they both acquire and cede land in the transnational context. In order to globally acknowledge for what was currently considered as abnormal cases, we model open access data about LSLAs extracted from the Land Matrix Initiative (LMI) open-access database into a network graph, and adapt an eigenvector based centrality method originally conceived for online social networks, namely *LurkerRank*, to identify and rank anomalous profiles in the land trade market. We take into account three different network snapshots: a *multi-sector* network (including all the transnational deals in the LMI database), and three networks referring to specific investment sectors (*agriculture*,*mines* and *biofuels*). Experimental results show that emerging economies (e.g., China and Malaysia) play a central role in the land trade market, by creating alternative dynamics that escape the classic North/South one. Our analyses also show how African countries that are often seen as targets of land trade transactions in a specific sector, may often acquire foreign land in the context of investments in the same sector (i.e., Zimbabwe for biofuels and the Democratic Republic of Congo for the mining sector).

## 1 Introduction

Large-scale national and transnational commercial land transactions, or Large-Scale Land Acquisitions (LSLAs) [1], are a global and consolidated phenomenon that plays an important role in basically every land-based business, including agriculture, forestry, mining, tourism, renewable energy, and many more. Even though such phenomenon has long been noticed, dating back (at least) to colonial and post-colonial business dynamics, a sudden increase of such kind of deals has been witnessed in recent years [2]. A major reason can be found in the long lasting impacts on the global markets of the worldwide financial and food crisis that took place in 2007/2009 [3] (e.g., the increase in food prices, that in turn resulted in an important decrease of food security parameters in several countries). A major point of discussion about this controversial phenomenon is to what extent these large scale investments have a positive impact on the economies of the target countries. In fact, some reference their potential to leverage valuable economic opportunities [4], while on the other side of the coin there exists a significant risk of corruption and impact on indigenous people’s livelihoods and habits (e.g., loss of land, marginalization) [5–7].

Despite the central importance of LSLAs in the global economic scenario [8], obtaining solid and trustworthy data about land deals is not straightforward. Among the many reasons, we can cite the fact that in many countries land deals may be under-regulated by public policies [9] (e.g., official procedures may not exist or be voluntary and non-binding) and that official and unofficial (e.g., NGOs) information sources may often be inconsistent, and in any case inaccurate with respect to the actual situation [10]. The main source of information about LSLAs, which is also the one used in this work, is the database built a decade ago by the Land Matrix Initiative (LMI) [11]. The LMI is a consortium of research and development partners that started collecting land-related data back in 2009, integrating information from heterogeneous sources, such as government data, public press, scientific publications and voluntary contributions from specific individuals or institutes. In database, the threshold for a land acquisition to be considered a “large scale” one is to involve more than 200 hectares of leased or sold land [12]. While the original focus of the LMI was on agricultural international investments, the database now includes national and transnational deals concerning also many other investment sectors, such as mining and forestry.

The relations among the countries involved in LSLAs are often deriving from ancient colonial power, so that the predominant dynamic of the land trade market is certainly the one that reflects the power asymmetry between Global North and the Global South. A notable example is represented by G20 countries investing in southern ones (e.g., Sub-Saharan Africa, Latin America, South-eastern Asia). More generally, it has been shown that LSLAs are often related to weak governance systems and overlapping land rights, and that rich investor countries target poorer economies with abundant land and water resources [13, 14].

However, recent research work based on complex network analysis [15, 16] confirmed that other important dynamics exist in the global land trade market, such as a South-South one (e.g., Latin American countries investing in Africa), and the one involving emerging economies, such as the so-called BRICS countries (Brazil, Russia, India, China and South Africa). More specifically, in [16] we defined the *LSLA-score*, a topology-based measure proportional to the ratio between sold and acquired land for each country, which enables the ranking of countries based on their investing/target role in the land trade network. Low values of *LSLA-score* correspond to *investing* countries, while higher ones to *target* countries. It was found that, while extreme values of the *LSLA-score* characterize well-known dynamics, i.e., investors located in the Global North (low values) and target countries located in the Global South (high values), medium ones often correspond to emerging economies (e.g., Brazil, South Africa).

**Fig 1:**
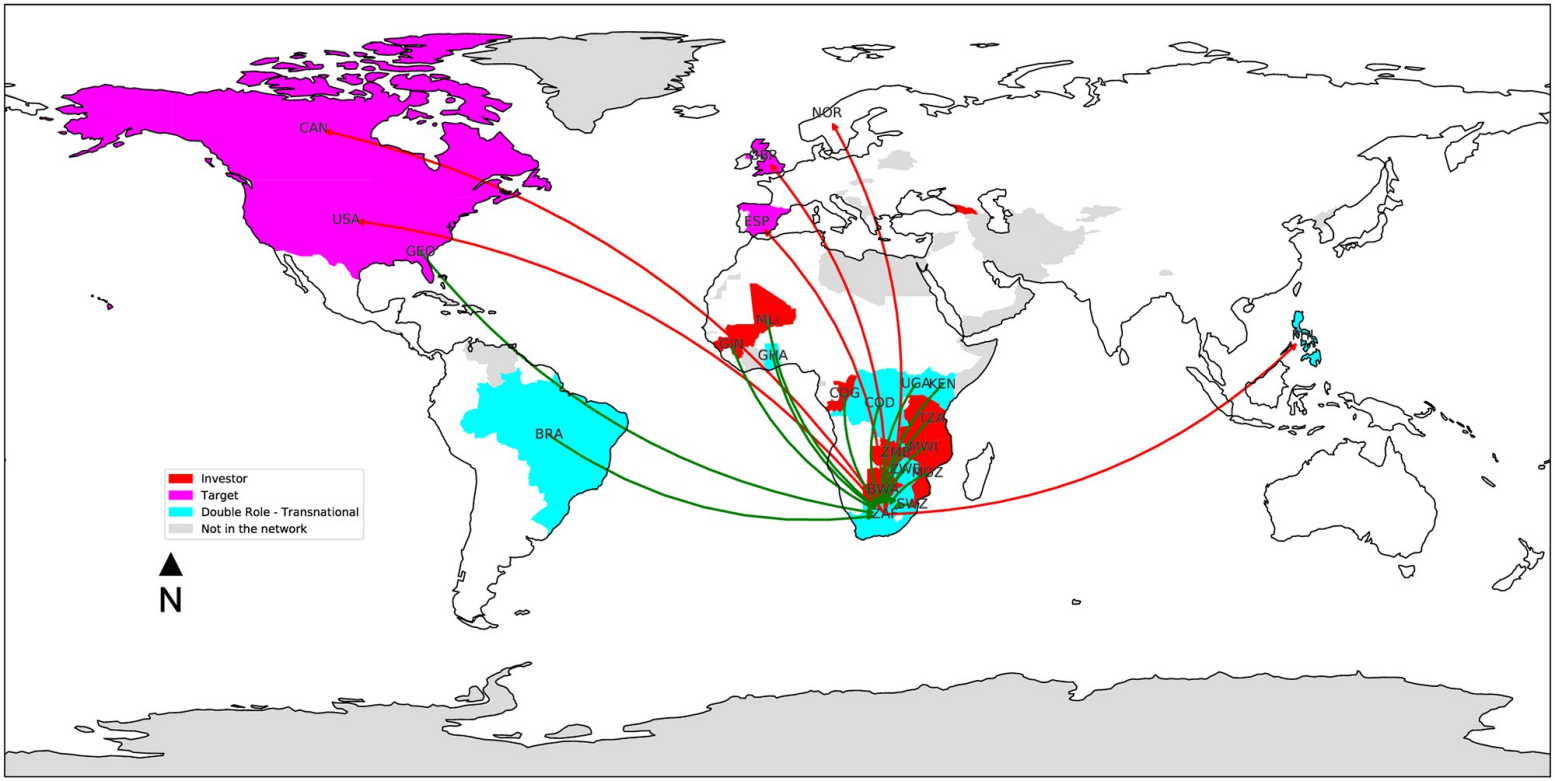
Focus on the ego network of South Africa. Deals seeing it as the target country (red edges) refer to investments in the sectors of food crops, renewable energies and livestock. On the other hand (green edges), South African companies invests in transnational deals (mainly in other African countries) in the same sectors, plus in different ones such as biofuels, tourism and mining.

While these countries tend to have an *investor* profile, they are often involved as target countries in several deals. Taking the transnational deals involving South Africa as case in point (Fig 1), it can be noted that the profile of this country is rather skewed towards an investor role, i.e., companies based in South Africa are involved (alone or associated to other investors) in deals for a total of about one million hectares of foreign land. However, at the same time, about ten thousands of hectares of South African land are acquired by foreign countries, i.e., six transnational deals involving companies from six different countries (USA, Canada, Spain, Norway, UK and Philippines). Moreover, it is interesting to note how these deals take place in sectors that at the same time see South Africa as a transnational investor, i.e., food crops (Zambia, Mozambique, Tanzania, Malawi, Georgia) and livestock (Swaziland, Nigeria).

The aim of this work is to perform a deeper investigation on this phenomenon, that can be considered an anomaly in the land trade market, i.e., less than 25% of the countries included in the transnational land trade network are characterized by this double investor/target profile. To this purpose, we will model open access data about LSLAs extracted from the Land Matrix Initiative database (https://landmatrix.org) into a network graph, and adapt an eigenvector based centrality method originally conceived for online social networks, namely *LurkerRank* [17–19], to identify and rank anomalous profiles in the land trade market. While the effectiveness of complex network analysis techniques on the analysis of the land trade market has already been confirmed by our previous works on such topic [15, 16], the scenario we study in this work requires a methodology with peculiar characteristics. In particular, we need a method able to capture at the same time the centrality of a country in the land trade market, and the entity of the acquired/sold land asymmetry representing the anomaly (i.e., overconsumption principle). To this purpose, we resorted to *LurkerRank*, because previous literature [17–19] has already shown how it largely outperforms competing methods for centrality problems based on the *overconsumption* principle (e.g., PageRank [20], alpha-centrality [21] and Fair-Bets [22]). Note also how *LurkerRank* has been proven to be robust and flexible to different analysis tasks (e.g., learning to rank [23]) and complex network models, such as multilayer networks [23, 24]. As will be discussed in detail in Section 3, the principles on which *LurkerRank* is based on make it perfectly suitable for the task at hand. As a consequence, we are confident that developing different methodologies of network analysis is not needed here. The original contributions of our work can then be considered threefold:

1. From a thematic point of view, this is the first work presenting a computational approach to the problem of land trade anomalies in the global market.
2. From a methodological point of view, to address the above problem, we propose a well-principled formulation based on eigenvector-analysis theory to develop a ranking method for countries in the land trade market.
3. Moreover, we identify and leverage a previously unexplored analogy between trading criteria and the overconsumption principle, which enables us to profitably exploit a well-established methodology in social network analysis and originally adapt it to the new context of land trade market.

For our experimental evaluation, we take into account three different network snapshot: a *multi-sector* network (including all the transnational deals in the LMI database), and three networks referring to specific investment sectors (*agriculture*,*mines* and *biofuels*). Results show that *LurkerRank* was able to correctly identify and rank countries showing an anomalous behavior on the land trade market in different investment sectors, while simpler indicators (i.e., the *LSLA-score*) are not suitable for this task. Our qualitative analysis underlines how emerging economies play a central role in the land trade market, by creating alternative dynamics that escape the classic North/South one. These emerging economies include, as expected, the BRICS countries (with China assuming a pivotal role), but also other emerging markets like Malaysia. We also showed how African countries that are often seen as targets of land trade transactions in a specific sector, may often acquire foreign land in the context of investments in the same sector, i.e., Zimbabwe for biofuels and the Democratic Republic of Congo for the mining sector.

## 2 Background knowledge

In this section, we will discuss preliminary knowledge needed for a complete understanding of the methodologies adopted in this work to identify and rank anomalies in the land trade market. We will first introduce the Land Matrix database, then we will discuss the network modeling choices, and finally we will briefly introduce the *LurkerRank* method.

### 2.1 The Land Matrix database

The networks used in this work have been modeled upon open access data extracted from the Land Matrix Initiative database. The snapshot of the database used for this work has been downloaded on March 17th, 2022, using the *Deals* page of the official website (https://landmatrix.org/list/deals/). Two filters have been applied: *Scope* of the deal has been set to be *Transnational* (since for the current analysis we do not need to take into account national ones), and *Implementation status* to be *In operation (production)* (in order to take into account only the deals that are currently active, and discard the abandoned, concluded and not yet started ones). Please note that, even though the LMI database represents the most comprehensive information source about LSLAs, it is, for the reasons discussed in the introduction to this work, not exhaustive. By consequence, some biases in the data cannot be avoided [25] and our results and analyses reflect current accessible data. Nevertheless, like in our previous network analysis based study on LSLAs [16], we make the hypothesis that such biases do not have a major impact on a global analysis like the one carried out in this work.

Main characteristics of the raw data are summarized in Table 1. Based on the *Intention of investment* attribute of the LMI database, we extract four different networks corresponding to different investment sectors in the land trade market:

- the *multi-sector* network, including all the transnational deals, regardless of the associated intention of investment;
- the *agriculture* network, obtained by selecting the deals having an *Intention of investment* associated to the macro-domain of *Agriculture* on the official website, i.e., *Biofuels*, *Fodder*, *Food crops*, *Agriculture unspecified*, *Livestock*, *Non-food agricultural commodities*.
- the *biofuels* network, obtained by selecting deals having *Biofuels* as *Intention of investment*;
- the *mines* network, obtained by selecting deals having *Mining* as *Intention of investment*.

**Table 1:** Characteristics of the Land Matrix Initiative datasets (as of 17 March 2022). The total involved land refers to the total size of deals currently in operation for each dataset.

*Dataset*	*multi-sector*	*agriculture*	*mines*	*biofuels*
*#deals (in operation)*	2312	1503	197	228
*total involved land (ha)*	74,013,888	24,975,905	6,390,354	5,698,318

Note that the *multi-sector* network includes the three other networks, and that *biofuels* is a subnetwork of *agriculture*. We judge the *biofuels* domain worth of a specific investigation, even if already included in the *agriculture* network, as it concerns an investment sector related to many hot topics, such as fossil fuel availability and renewable energies; moreover, it can be noted how the resulting network has the same order of magnitude of the other one referring to another specific investment sector (i.e., *mines*). *The Python code, as well as the original Land Matrix data and the land trade networks used in the context of this work are publicly available online at*: (https://gitlab.irstea.fr/roberto.interdonato/lsla-networkanalysis).

### 2.2 Modeling the land trade network

We define the land trade network as a directed graph *G* = (*V*, *E*, *w*), where *V* is a set of nodes, *E* is a set of edges, and w:E↦R is an edge weighting function. Each node *v* ∈ *V* represents a country involved in at least a transnational deal in the Land Matrix, i.e., as target country and/or as country of registration/origin of an investor (for this reason, in the following discussions, we may refer to a node while implicitly referring to the corresponding country). Given two nodes *u*, *v* ∈ *V*, edge (*u*, *v*) exists in *G* if there is at least one deal in the LMI database such that a company based in *v* is involved as investor in a deal where *u* is the target country. Simplifying, we say that an edge (*u*, *v*) exists when *v* acquires land from *u*. Edge weights are computed as the total size (in hectares) associated to the deals involving *u* as target and *v* as location of the investor. Note that the network modeling is defined under the same principles described in our previous work [16], with a main difference being the fact that in this case we use an inverse orientation for the edges, i.e., edge is oriented from the target country to the investing one. The idea is that the direction of an edge follows that of the ownership of the land, i.e., from the target country (original owner) to the investor/acquiring one. This choice is made in compliance to the original semantics of the *LurkerRank* method, as will be pointed out while describing the methodology in Section 3.

In presence of multiple investing companies from different countries on a same deal, at modeling time we consider the deal as belonging equally to each country with the same (total) deal size. As pointed out in [16], while this assumption introduces an overestimation in the total edge weights, it is, in our opinion, the most reasonable one in our analysis scenario. Similarly, deals reporting multiple values in the *Intention of investment* attribute have been equally considered in all associated networks.

### 2.3 The *LSLA-score*

Here, we briefly recall one of the main contributions of our previous work about Large Scale Land Acquisitions [16], i.e., the *LSLA-score*. The aim of the *LSLA-score* is to provide a rank of the countries based on their investor/target role on the global land trade market. The score is proportional to the ratio between the total surface of sold and acquired land for each country, as reported in the Land Matrix database. Note that we refer to *sold* land with a wide meaning, which includes contracts of different nature (e.g., leasing, renting, etc.). The *LSLA-score* *s* for each country *u* in the land trade network can be formally defined as:

$$\begin{array}{c} s\left(u\right)={\text{log}}_{10}\;(\frac{1+\sum_{(u,v)\in E}w\left(u,v\right)}{1+\sum_{(v,u)\in E}w\left(v,u\right)}) \end{array}$$ (1)

In Eq 1 the numerator corresponds to the total surface of land sold by *u* to foreign countries, while the denominator corresponds to the total surface of land that *u* acquires from foreign countries. The values of *s*(⋅) are Laplace add-one smoothed in order to prevent zero or infinite ratios. By definition, low values of *LSLA-score* will correspond to *investing* countries, while higher ones to *target* countries. The logarithmic scaling is added with the aim to make the score distribution smoother. Note that Eq 1 has been adapted, with respect to the original definition in [16], to be compliant with the semantic of the edge orientation used in this work.

## 3 Ranking anomalies with the LurkerRank method

In order to identify and rank anomalies in the land trade networks, we resort to a concept originally conceived in the context of Online Social Network (OSN) analysis, that of *lurker*, by exploiting the *LurkerRank* (LR) method [18]. A lurker can be defined as a rather silent user, who gains benefit from others’ information and services without giving back to the OSN through tangible actions. LR is an eigenvector-centrality-based ranking method that exploits the topological characteristics that derive from such participation inequality in order to identify and rank lurkers in a network graph.

The three main principles underlying the original *LurkerRank*, formulated in reference to the OSN domain, can be summarized as follows [18]:

- **P1**. Overconsumption, i.e., the excess of information-consumption over information-production.
- **P2**. Authoritativeness of the information received, i.e., the valuable amount of information received from its in-neighbors.
- **P3**. Non-authoritativeness of the information produced, i.e., the non-valuable amount of information sent to its out-neighbors.

Our hypothesis is that these principles can be adapted to the context of a land trade network, where the flow of acquired land among countries can be taken into account for the ranking process instead of that of information among users in an OSN. By consequence, for our task of identification and ranking of anomalies in the land trade market, we revise the above principles as follows:

- **P1**. Uneven investor/target role. In the context of LSLAs, the presence of a double role is an indicator of anomaly. The entity of the anomaly can be considered greater if a country with a strong profile on one of the two roles, also assumes the other one in some deals (e.g., country with a strong investor profile that also appears as a target, or vice versa).
- **P2**. Amount of land that a country acquires from ones showing a stronger investor profile. The fact that a country acquires land from one that has a more consolidated role of investor than its own can be considered an indicator of anomaly.
- **P3**. Amount of land that a country sells to ones showing a stronger target profile. The fact that a country sells land to one that mainly assumes the role of target can be considered an indicator of anomaly.

Summarizing, **P1** focuses on the presence of a double investor/target role, while **P2** and **P3** build upon such asymmetry to quantify the anomalous activities on the land trade market. Note that, in the formulation of **P2** and **P3**, the concept of rich/poor country is used in relation to its activity on the land trade market, and not as a general indicator to its financial state (even if the former can be often considered as a proxy to assess the latter).

The above lurking principles can hence be instantiated into a full ranking model that takes into account such different aspects in order to produce a score for each node in the network [17, 19]. For a node *v* ∈ *V*, the contribution of its in-neighborhood to the score (corresponding to the aspect described in **P2**), namely $L_{\text{in}}\left(v\right)$, can be defined as follows:

$$\begin{array}{c} L_{\text{in}}\left(v\right)=\frac{1}{|N^{out}\left(v\right)|}\sum_{u\in N^{in}\left(v\right)}w\left(u,v\right)\frac{|N^{out}\left(u\right)|}{|N^{in}\left(u\right)|}L_{\text{in}}\left(u\right) \end{array}$$ (2)

Where w(u,v) is the weight of the edge (u,v), and the *N*^*in*^(⋅) and *N*^*out*^(⋅) are set functions corresponding, respectively, to the in-neighborhood and the out-neighborhood of a node. Note that the cardinality values are Laplace add-one smoothed, in order to prevent zero or infinite ratios. Similarly, the contribution of a node’s out-neighborhood $L_{\text{out}}\left(v\right)$ (corresponding to the aspects described in **P3** it is defined as:

$$\begin{array}{c} L_{\text{out}}\left(v\right)=\frac{|N^{in}\left(v\right)|}{\sum_{u\in N^{out}\left(v\right)}\left|N^{in}\left(u\right)\right|}\sum_{u\in N^{out}\left(v\right)}w\left(v,u\right)\frac{|N^{in}\left(u\right)|}{|N^{out}\left(u\right)|}L_{\text{out}}\left(u\right) \end{array}$$ (3)

The three principles can then be integrated into a unified lurking model:

$$\begin{array}{c} L_{\text{inout}}\left(v\right)=(L_{\text{in}}\left(v\right))(1+L_{\text{out}}\left(v\right)) \end{array}$$ (4)

In [17, 19] this model is called *in-out-neighbors-driven lurking*, i.e., in order to distinguish it from *in-neighbors-driven* and *out-neighbors-driven lurking* models taking into account just one of the two aspects. For this work, we will exploit this model in order to simultaneously take into account the three principle while ranking anomalies in the land trade market.

The final *LurkerRank* algorithm consists in a complete specification of the lurker ranking model in terms of *PageRank* [20], i.e., a well-known eigenvector centrality approach. The PageRank-based ranking equation for the *in-out-neighbors-driven LurkerRank* score for any node *v*, can be defined as follows:

$$\begin{array}{c} L_{\text{inout}}\left(v\right)=d\left(L_{\text{inout}}\left(v\right)\right)+\left(1-d\right)p\left(v\right) \end{array}$$ (5)

where *p*(*v*) denotes the value for *v* in the personalization (or teleportation) vector, which is by default set to 1/|*V*|, and *d* is a damping factor ranging within [0, 1], usually set to 0.85. In the following, we will refer to this formulation simply as *LurkerRank*.

Note that the classic PageRank algorithm [20] cannot be directly applied to define the lurking/anomalous behavior described by the three principles, since it assumes that node relations follow the flow of influence propagation, which is related to the *amount of information a node produces* (e.g., amount of sold land in our scenario). By contrast, lurking behaviors build on the *amount of information a node consumes* (e.g., amount of acquired land in our scenario). In terms of topology, if an investor located in country *v* acquires a certain amount of land from country *u*, then *v* is likely to benefit from the resources located/produced in country *u*. It is according to this concept, which is fundamental to obtain a proper ranking from *LurkerRank*, that in this work we use an “inverted” topology with respect to the land trade network modeling presented in [16].

It can be observed how, while *LurkerRank* is devised to take into account the three principles, the *LSLA-score* just takes into account **P1**, by measuring an eventual uneven investor/target role (cf. Sec. 2.3). This makes the *LSLA-score* unsuitable to correctly rank anomalies in the land trade market, as will be analyzed in detail in Sec 4.

**Fig 2:**
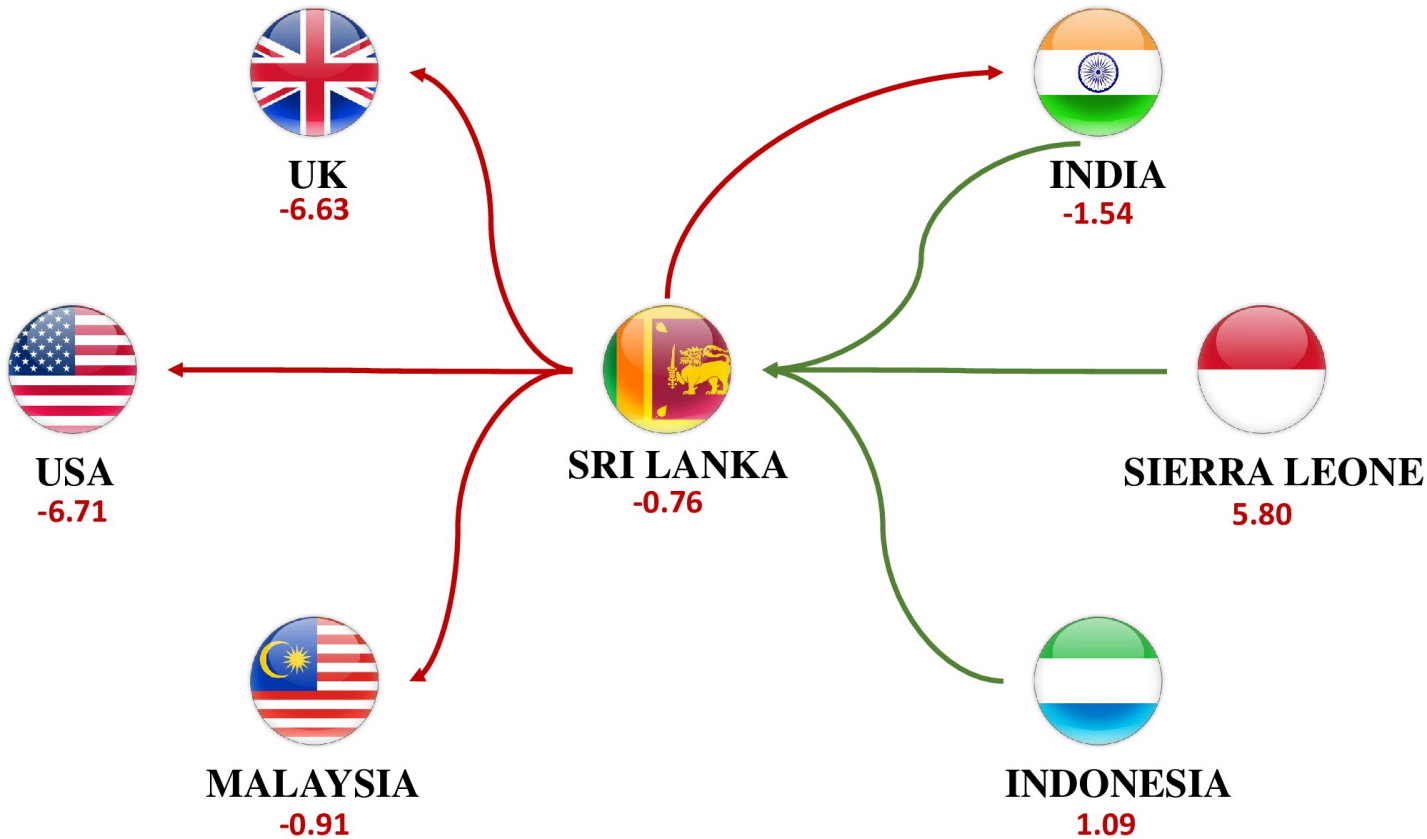
Ego network of Sri Lanka in the *lurking oriented* land trade network. Incoming links (in green) represent deals that involve companies located in Sri Lanka as investors, outgoing links (in red) represent deals where Sri Lanka is the target country. Below the name of each country, we report its *LSLA-score* (recall that low values of *LSLA-score* correspond to countries having an investor profile, while high values correspond to countries showing a target profile).

**Running example**. To provide an intuition of how *LurkerRank* works, we can take as case in point the LSLAs associated to the country of Sri Lanka. In Fig 2 we report the ego network of the node associated to Sri Lanka in the *multi-sector* land trade network. Incoming links (in green) represent deals that involve companies located in Sri Lanka as investors, outgoing links (in red) represent deals that see Sri Lanka as the target country. Below the name of each country, we report its *LSLA-score*, calculated as defined in [16]: recall that low values of *LSLA-score* correspond to countries having an investor profile, while high values correspond to countries showing a target profile. Investors from UK, USA, India and Malaysia acquire land from Sri Lanka (mainly for food crops related investments), while investors based in Sri Lank acquire land from Indonesia (Food Crops), Sierra Leone (Biofuels, Food Crops, Renewable Energy) and India (Textile Industry). Resorting to the three previously defined principles, we can easily observe:

- Presence of a double investor/target role (**P1**). While the role is not strongly uneven in terms of number of relations, it can be defined as such in terms of total surface (i.e., around 140k hectares of acquired land vs around 20k hectares of sold land).
- Presence of a deal where land is acquired from a country with a stronger investor profile (i.e., India) (**P2**). Note that India not only has an *LSLA-score* lower than Sri Lanka (−1.54 vs −0.76), but is also regarded as one of the major emerging economies worldwide (i.e., the so-called *BRICS* countries, cf. Sec. *Introduction*).
- Presence of a deal where land is sold to a country that does not have a strong investor profile (i.e., Malaysia) (**P3**). Even if Malaysia has an *LSLA-score* slightly lower than Sri Lanka (i.e., −0.91 vs −0.76), it does not have a strong investor profile like UK (−6.63) or USA (−6.71).

By consequence, we can expect *LurkerRank* to rank Sri Lanka among the top anomalies, as it correctly does by ranking it at the 5-th position out of 36 countries showing a double role in the *multi-sector* network (cf. Table 3). Note how its raking position decreases to 12 in the *agriculture* network, where the land acquisition from India, that can be easily considered as the most “anomalous” one in the ego network, is not taken into account (since it is associated to the industry sector).

## 4 Experimental results

### 4.1 Structure of the land trade networks

Table 2 reports on the structural characteristics of the four land trade networks we extracted from the LMI database: *multi-sector*, *agriculture*, *mines* and *biofuel*. All measures refer to the directed version of the graph, except for the average clustering coefficient (*avg_cc*) and average path length (*avg_path_length*) which are computed on the undirected graph. Note that the latter is not available for the *agriculture* network since it is not weakly connected, due to a disconnected component corresponding to the Barbados and Guyana countries (the average path length associated to the largest connected component subgraph is reported in parentheses). Note how *multi-sector* and *agriculture* share similar structural characteristics, as well as *mines* and *biofuel*. More in detail, some structural characteristics are clearly related to the narrowness of the associated investment sector: networks covering a wider scope (i.e., *multi-sector* and *agriculture*) are naturally denser, and in particular they have smaller average path length and higher average clustering coefficient. By contrast, some characteristics are common to all the four networks, such as an extremely low percentage of reciprocal edges (even 0 in the case of *mines*) and the tendency of being slightly degree-disassortative (more evident in specific sectors like *mines* and *biofuels*). Recall that degree assortativity measures the tendency of a node to connect with similar ones in terms of degree, which in our context corresponds to observing land trade deals among countries with similar profiles. We can observe how these structural characteristics confirm the hypotheses behind this work: deals among countries with similar profiles (e.g., like Sri Lanka and Malaysia in the example reported in Fig 2) and reciprocal edges (e.g., like Sri Lanka and India in the same example) are extremely rare and should be regarded as anomalies.

**Table 2:** Structural characteristics of the land trade networks. All measures refer to the directed version of the graph, except for *avg_cc* and *avg_path_length* which are computed on the undirected graph. Note that the latter is not available for the *agriculture* network since it is not weakly connected (value associated to the largest connected component is reported in parentheses).

*network*	*#nodes*	*#edges*	*reciprocity*	*avg_path_length*	*avg_cc*	*transitivity*	*assortativity*
*multi-sector*	152	733	0.01	2.59	0.17	0.07	-0.14
*agriculture*	144	539	0.01	∞(2.79)	0.1	0.05	-0.11
*mines*	76	141	0	3.15	0.06	0.05	-0.23
*biofuels*	87	140	0.01	3.78	0.06	0.02	-0.17

**Fig 3:**
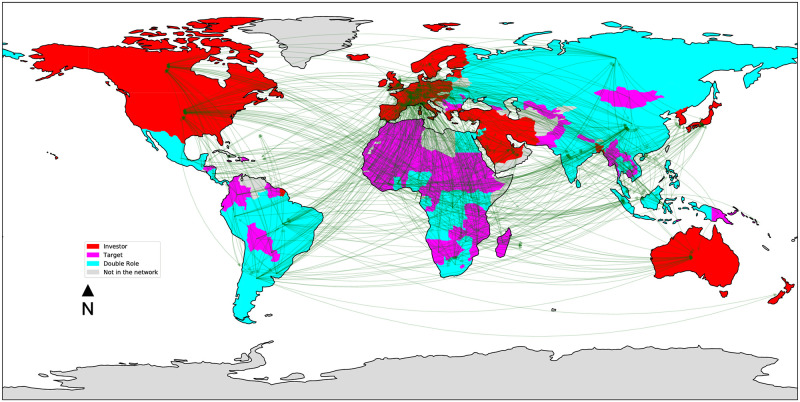
The *multi-sector* land trade network, where countries are colored based on their role: Source nodes/pure investors (red), sink nodes/pure targets (magenta), double role countries (cyan). Countries colored in grey are absent from the network.

**Fig 4:**
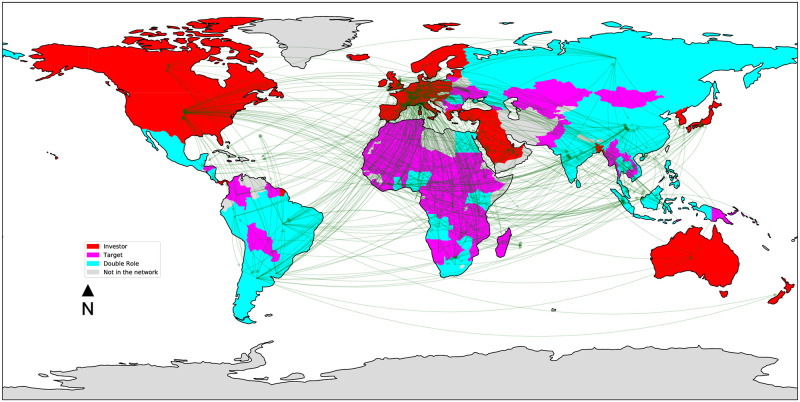
The *agriculture* land trade network, where countries are colored based on their role: Source nodes/pure investors (red), sink nodes/pure targets (magenta), double role countries (cyan). Countries colored in grey are absent from the network.

**Fig 5:**
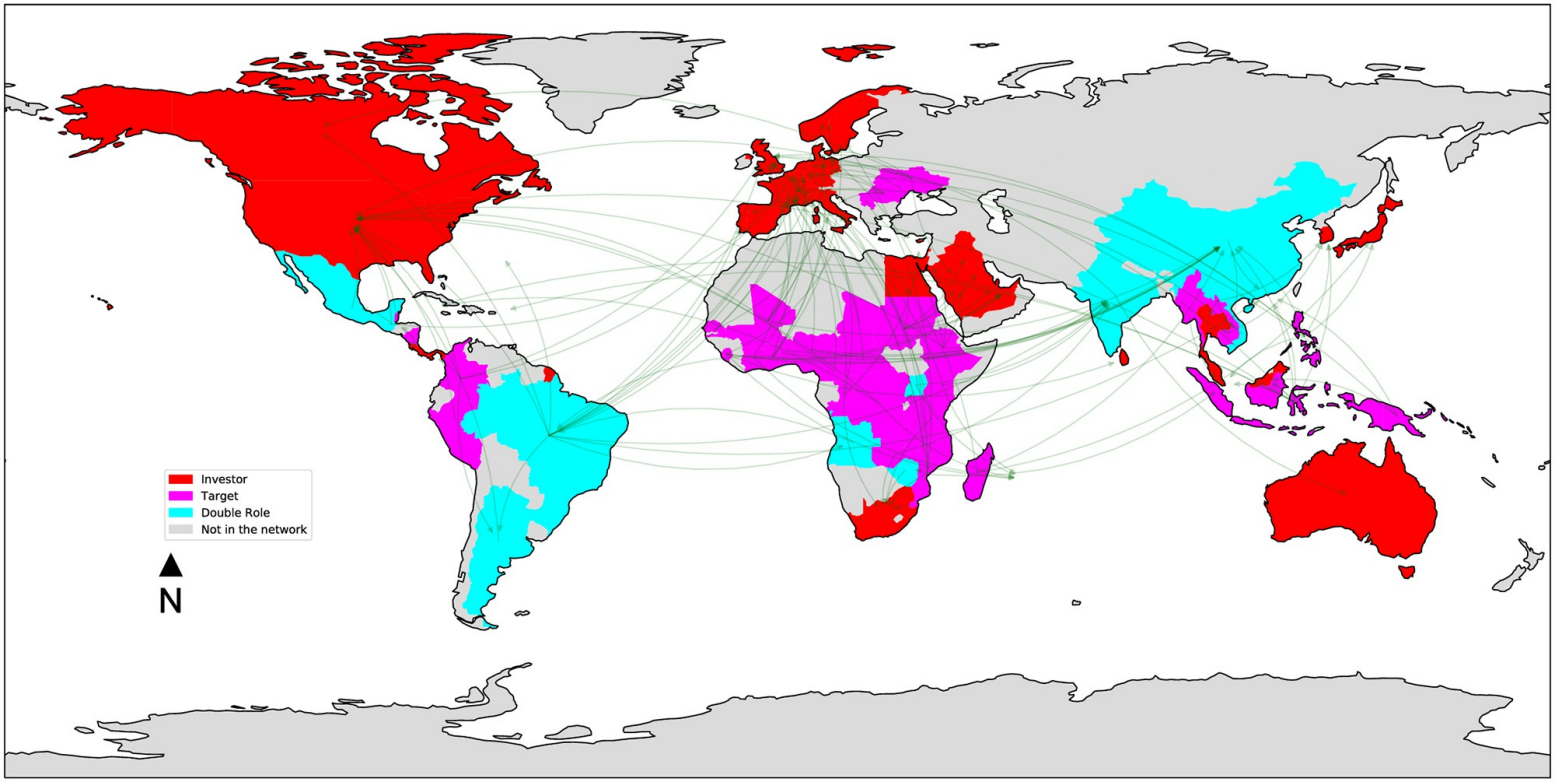
The *biofuels* land trade network, where countries are colored based on their role: Source nodes/pure investors (red), sink nodes/pure targets (magenta), double role countries (cyan). Countries colored in grey are absent from the network.

**Fig 6:**
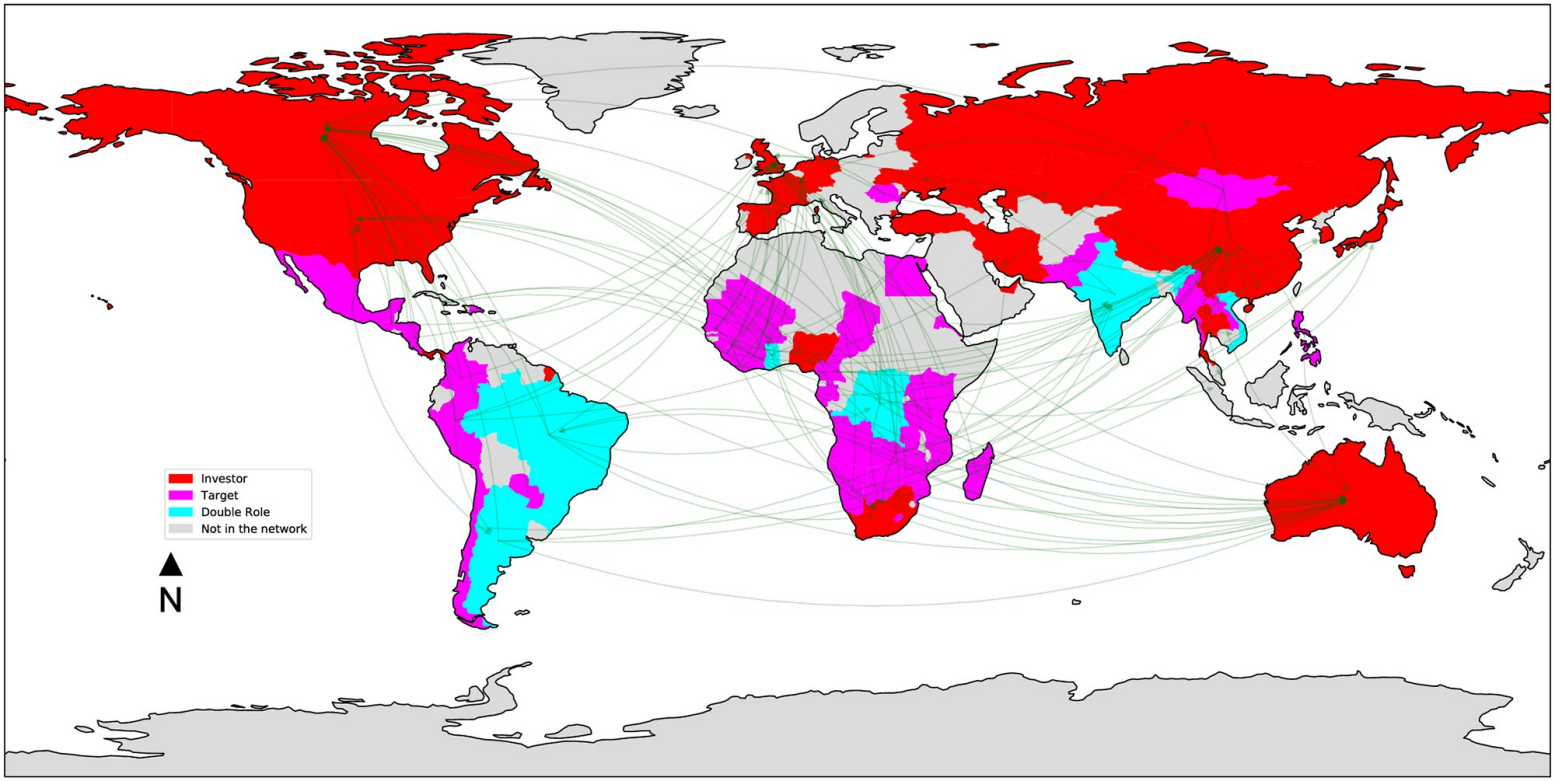
The *mines* land trade network, where countries are colored based on their role: Source nodes/pure investors (red), sink nodes/pure targets (magenta), double role countries (cyan). Countries colored in grey are absent from the network.

Figs 3–6 depict the four land trade networks, with an emphasis on the role of each country, i.e., source nodes/pure investors (red), sink nodes/pure targets (magenta) and double role countries (cyan). Countries colored in grey are absent from the mining trade network. If we focus on double role countries, since they correspond to potential anomalies, it can be noted how the number of countries assuming this role decreases as the investment sector becomes more specific, i.e., a lower number of potential anomalies can be identified in narrow sectors such as *mines* and *biofuels*. In the *multi-sector* (Fig 3) and *agriculture* (Fig 4) networks we observe the absence of double role countries in North America, Europe, Middle East and the major part of Oceania, which are characterized by a massive presence of pure investors. Similar considerations can be drawn for the *biofuels* network (Fig 5), where it can be noted the absence of a main actor like Russia from the network, and the presence of fewer double role countries in Africa. Finally, the separation between Global North and Global South is extremely evident in the *mines* network (Fig 6), where potential anomalies can be observed only in South America, Africa, and Southern/Southeastern Asia.

### 4.2 Ranking of the anomalous land trade behaviors

Table 3 shows the top countries as ranked by *LurkerRank* on the four land trade networks, together with their ranking score (recall that the score reported in the table is the ranking score produced by *LurkerRank*, and it is not related to the *LSLA-score* described in Sec. 2.3). Sink (i.e., pure investors) and source (i.e., pure target) nodes are excluded from the ranking, in compliance with the fact that only double role countries are regarded as potential anomalies. It can be observed that *multi-sector* and *agriculture* share the same top-2 countries (Malaysia and China), even if the distance between their ranking scores is considerably higher in the second case. Note also that these two networks share 10 out of 15 top-ranked countries, even if with different positions in the ranking: this can be considered as an expected result, since investments in the agricultural sector represent the great majority of the deals reported in the Land Matrix database. China also appears as the top ranked country in the *biofuels* network, confirming how such emerging economy is still targeted by foreign investors, even though being investing since 2010 in different sectors, targeting worldwide countries. Some examples of investments involving Chinese companies are Russia, Guyana, Congo Democratic Republic and Mozambique for agriculture, Mali and Madagascar for biofuel and South-East Asia for forestry, with the latter being an area also targeted for mining investments together with several African countries. At the same time, China is targeted for several deals in the context of timber plantations, involving companies from Canada (Cathay Forest Products Corp.), Japan (Marubeni Co., Oji Paper Co Ltd), Finland (Stora Enso) and Singapore (Royal Golden Eagle International), that also appears, with a different company (Singbridge), in a deal about the construction of a modern agricultural food zone in the north-east of the country. Finally, we can point out that Chinese companies also invest in large timber plantations (i.e., the main resource for which China is a target country) in Laos and Cambodia. Focusing on Malaysia, a country that appears as the top ranked on two out of four networks, it is a massive investor, with its companies involved in more than 100 investing deals worldwide. Such deals mainly include biofuels, food crops and other agricultural investments in South-East Asia (mainly in Indonesia, but also in Laos, Cambodia and Papua New Guinea) and several African countries (Congo, Ghana, Ivory Coast, Liberia, Nigeria and Uganda) and forestry investments in Liberia and Russia. At the same time, Malaysia land is targeted by several foreign investors. The main examples is an Indian company (Avantha Group) investing in a timber plantation (again, a resource for which Malaysia invests in foreign land), that represents one of the largest deals in the entire database (more than 288k hectares). The large complex includes not only the trees, but also paper and plywood manufacturing facilities, biofuels and a 40% of land destined for conservation. Other examples include a palm oil related cultivation involving investors from USA (Archer Daniels Midland Company) and an agricultural land acquisition (producing Banana, Coconut and Oil Palm) involving a company from Denmark (United International Enterprises). Among the countries investing in Malaysia we can also find Japan, China, India (mainly for agricultural lands) and Netherlands (FACE Foundation investing in an eco-tourism/conservation project). We can easily state, based on this detailed qualitative overview about the land trade deals involving China and Malaysia, that *LurkerRank* was able to correctly place such countries at the top of the ranking.

**Table 3:** Top-ranked countries and their ranking scores obtained by *LurkerRank* in the *multi-sector*, *agriculture*, *biofuels* and *mines* networks. Sink (i.e., pure investors) and source (i.e., pure target) nodes are excluded from the ranking, in compliance with the fact that only double role countries are regarded as potential anomalies.

	*multi-sector*		*agriculture*		*biofuels*		*mines*	
*rank*	*country*	*score*	*country*	*score*	*country*	*score*	*country*	*score*
1	Malaysia	1.78E-01	Malaysia	9.57E-01	China	3.60E-05	India	1.79E-01
2	China	1.54E-01	China	2.51E-02	India	8.00E-06	Congo, D.R.	1.16E-02
3	India	6.90E-03	Mauritius	3.59E-03	Zimbabwe	1.00E-06	Brazil	2.29E-04
4	Chile	1.73E-03	India	2.41E-03	Argentina	0.00E+00	Argentina	1.44E-04
5	Sri Lanka	5.33E-04	Vietnam	4.63E-04	Vietnam	0.00E+00	Ghana	6.00E-06
6	Indonesia	4.66E-04	Indonesia	4.43E-04	Mexico	0.00E+00	Vietnam	0.00E+00
7	Argentina	3.77E-04	Argentina	4.03E-04	Guatemala	0.00E+00	-	-
8	Ghana	1.77E-04	South Africa	7.10E-05	Uganda	0.00E+00	-	-
9	Nigeria	1.15E-04	Brazil	6.70E-05	Angola	0.00E+00	-	-
10	Lithuania	8.20E-05	Russian Fed.	5.50E-05	Brazil	0.00E+00	-	-
11	Vietnam	7.20E-05	Ghana	5.30E-05	-	-	-	-
12	South Africa	2.50E-05	Sri Lanka	5.30E-05	-	-	-	-
13	Gabon	3.00E-06	Belize	1.10E-05	-	-	-	-
14	Mauritius	2.00E-06	Egypt	5.00E-06	-	-	-	-
15	Congo, D.R.	1.00E-06	Thailand	5.00E-06	-	-	-	-

Looking at Table 3, it’s easy to see how all the so-called BRICS countries (Brazil, Russia, India, China and South Africa) appear at least in a top-ranking, and all of them appear among the top-ranked countries in the *agriculture* network. This confirms our motivating hypothesis that these emerging economies are at the center of the main dynamics of the land trade market that can be considered “alternative” to the classic Global North vs Global South narrative. While from one side these countries have nowadays grown significant investments capacities, on the other hand they often have a scarce quantities of arable land and water to exploit, as well as increasing populations. For this reason, they often tend to address several resource needs by resorting to investments involving the acquisition of land in foreign countries.

When looking at more specific markets like *biofuels* and *mines*, we can discover the presence of several African countries among the top-anomalies. Observing the *biofuels* ranking, we can note the presence of Zimbabwe: while being a target country in two deals involving a South-African company (Tongaat Hulett Sugar South Africa Ltd.), on large land areas mainly exploited for the production of sugar cane and biofuels (that supply about two-thirds of the energy in the country [26]), it also appears as single investor (through the Rift Valley Corporation) in a deal regarding the acquisition of 150k hectares of land for the production of biofuels in Mozambique, which is one of the most promising African countries in terms of biophysical potential of agrofuel/biodiesel production from vegetable oils [27]. As regards the anomalies in the *mines* land trade market, it is the presence of the Democratic Republic of the Congo that pops up. The country is a target for several foreign investors in the mining domain (mainly China and Canada, but also UK, Australia, South Africa, Switzerland and Gibraltar) for the extraction of resources such as gold, cobalt and copper [28]. At the same time, a company based in the country (OKIMO—Offices de Mines d’Or de Kilo Moto) is investing, together with a South-African one (Anglo Gold Ashanti) in a gold mine in Brazil, in the area of Minas Gerais.

#### Efficiency of *LurkerRank*

Regarding the computational complexity of *LurkerRank*, similarly to other eigenvector centrality methods like PageRank [20], it is proportional to the number of edges in a network, i.e., O(|E|). This can be easily verified by looking at the execution times reported in Table 4, where the execution time on smaller networks like *mines* and *biofuels* is around 10 milliseconds lower than on the bigger ones (*multi-sector* and *agriculture*)(experiments were carried out on a MacBook Pro with M1 Processor and 16 GB of RAM).

**Table 4:** Execution time of *LurkerRank* (milliseconds) on the different land trade networks (mean and standard deviation over 50 runs).

*Dataset*	*multi-sector*	*agriculture*	*mines*	*biofuels*
*execution time (ms)*	48.5±4.3	45.0±4.0	35.3±4.6	38.4±4.7

### 4.3 Correlation between *LurkerRank* and *LSLA-score*

As a final test about the significance of the ranking produced by *LurkerRank*, we compare it to the ones obtained by using the *LSLA-score* (cf. Sec. 2.3). First, we computed the Pearson correlation coefficient between the ranking produced by *LurkerRank* on the *multi-sector* network, and the one obtained when using the *LSLA-score*. We focus on the *multi-sector* network for this first test since it includes a larger number of potential anomalies (36), making a ranking correlation test more significant. As a result, we obtain a negative correlation of 0.36, which is once again in line with our hypotheses and with the previously discussed results. From one side, we can tell, from the fact that correlation is not close to 1, that using an eigenvector-centrality based algorithm that takes into account the full network context (*LurkerRank*) indeed produces different results than resorting to a purely local metric (*LSLA-score*). At the same time, the fact that correlation is not close to zero is compliant with the fact that countries showing an anomalous behavior often belong to a specific part of the *LSLA-score* raking (the middle one), i.e., in consequence of the fact that pure investors are mainly located at the bottom of the *LSLA-score* ranking, while pure targets mainly correspond to high *LSLA-score* values.

To delve more into the differences between the two methodologies, in Table 5 we report the top-ranked countries by *LSLA-score* in the *multi-sector*, *agriculture*, *biofuels* and *mines* networks. Sink (i.e., pure investors) and source (i.e., pure target) nodes have been excluded from the ranking, so that only double role countries, corresponding to potential anomalies, are retained for the analysis. Note that, based on the results of previous analyses, an *investor* orientation of the rank has been chosen (i.e., lower scores first) in order to emphasize similarities with the results produced by *LurkerRank*. Recall that the *LSLA-score* is proportional to the ratio between the total surface of sold and acquired land for a specific country, so that we can say that it just takes into account **P1**, by measuring an eventual uneven investor/target role (cf. Sec. 3). Moreover, note that countries that show an uneven situation skewed toward different roles, will be found on the opposite sides of the ranking: if the quantity of acquired land is greater than that of sold one, they will correspond to lower scores, vice versa countries that mostly assume a target role will get higher scores. This is not a desirable property in the task we are addressing in this work, because both situations may be indicators of anomaly, so that they should both contribute to increase the rank of a country (as they correctly do in the case of *LurkerRank*). At a first glance, we can easily note some similarities: the top ranked country in the four networks is the same as in Table 3, top-3 ones in the case of *agriculture*. As already mentioned, this is purely due to their strongly unbalanced ratio of acquired and sold land, and not to the anomalous nature of their behavior on the land trade market. In fact, at the same time, we can also observe major differences in the rest of the rankings with respect to the ones produced by *LurkerRank*. Regarding *multi-sector*, China, that was identified as one of the major anomalies, being ranked #2 by *LurkerRank* (with a score two orders of magnitude greater than that of the country ranked #3), has been ranked #3 by *LSLA-score*, equally to South Africa (ranked #12 by *LurkerRank*) and Thailand (not among the top-ranked countries by *LurkerRank*). Conversely, Mauritius (#14 by *LurkerRank*) is now ranked #2: by inspecting its situation, we can easily state that it is basically an investor country (investing in several other African countries in different sectors, for a total of nearly 200*k* hectares of land), with a single deal seeing it as a target country, involving a company from Singapore investing in 500 hectares of rice cultivation. It is easy to see then that its *LSLA-score* rank purely derives from the strong unbalance between acquired and sold land, and not from an anomalous behavior. Several similar examples can be named also for the other networks. To stick with situations that we previously analyzed in detail, we can cite Zimbabwe (#3 for *LurkerRank* in *biofuels*) ranked #8 by *LSLA-score*, and Congo (#2 for *LurkerRank* in *mines*) that is at the bottom of the ranking produced by *LSLA-score*. We can then conclude, from the results of this last analysis step, that a specific algorithm (*LurkerRank*) is actually needed to rank anomalies in the land trade market, while just selecting the double role countries from the ranking produced by *LSLA-score* would not be enough to fully assess the entity of their anomalous behavior in such a complex context.

**Table 5:** Top-ranked countries by *LSLA-score* in the *multi-sector*, *agriculture*, *biofuels* and *mines* networks. Sink (i.e., pure investors) and source (i.e., pure target) nodes are excluded from the ranking, in compliance with the fact that only double role countries are regarded as potential anomalies. An *investor* orientation of the ranking has been chosen (i.e., lower scores first) in order to emphasize similarities with the results produced by *LurkerRank*.

	*multi-sector*		*agriculture*		*biofuels*		*mines*	
*rank*	*country*	*LSLA-score*	*country*	*LSLA-score*	*country*	*LSLA-score*	*country*	*LSLA-score*
1	Malaysia	-1.09	Malaysia	-1.31	China	-0.88	India	-0.37
2	Mauritius	-0.88	China	-1.22	Vietnam	-0.54	Vietnam	0.00
3	South Africa	-0.75	Mauritius	-0.88	India	-0.26	Ghana	0.30
4	China	-0.75	Thailand	-0.85	Mexico	0.00	Argentina	0.48
5	Thailand	-0.75	Vietnam	-0.80	Argentina	0.00	Brazil	0.75
6	Lithuania	-0.54	South Africa	-0.73	Angola	0.00	Congo, D. R.	0.93
7	India	-0.51	India	-0.72	Uganda	0.12	-	-
8	Panama	-0.40	Sri Lanka	-0.26	Zimbabwe	0.18	-	-
9	Sri Lanka	-0.34	Zimbabwe	0.00	Guatemala	0.30	-	-
10	Vietnam	0.06	Mexico	0.00	Brazil	0.90	-	-
11	Belize	0.00	Belize	0.00	-	-	-	-
12	Jordan	0.00	Costa Rica	0.18	-	-	-	-
13	Chile	0.10	Egypt	0.19	-	-	-	-
14	Zimbabwe	0.16	Peru	0.20	-	-	-	-
15	Egypt	0.22	Guatemala	0.22	-	-	-	-

## 5 Conclusions

In this work, we focused on the phenomenon of Large-Scale Land Acquisitions (LSLAs), by performing a detailed analysis of the anomalous behaviors that can be identified in the transnational land trade market. We modeled open data about LSLAs, extracted from the Land Matrix Initiative database, in an oriented network graph, and proposed the adaptation of an eigenvector based centrality method originally conceived for online social networks, namely *LurkerRank*, to identify and rank anomalous profiles in the land trade market. Analyses performed on the entirety of the available land trade deals (*multi-sector* network) and on specific sectors of investment (*agriculture*, *mines* and *biofuels* networks) showed how emerging economies (e.g., China and Malaysia) play a central role in the land trade market, by creating alternative dynamics that escape the classic North/South one. Our analyses also show how African countries that are often seen as targets of land trade transactions in a specific sector, may often acquire foreign land in the context of investments in the same sector (i.e., Zimbabwe for biofuels and the Democratic Republic of Congo for the mining sector).

As future work, we plan to integrate detailed data about the investors (e.g., ownership trees of the companies, details about the participation of different stakeholders) in order to model a land trade network at a finer grain, that will allow us to represent with more precision the flow of investments, thus getting more insights about the different dynamics that animate the land trade market. Regarding the methodology, we plan to integrate the time dimension (e.g., date of start and/or implementation of the land acquisition) in order to analyze how the behavior of the countries evolves over different periods of time.
